# *In vitro* efficacy of next-generation dihydrotriazines and biguanides against babesiosis and malaria parasites

**DOI:** 10.1128/aac.00423-24

**Published:** 2024-08-13

**Authors:** Pratap Vydyam, Meenal Chand, Shalev Gihaz, Isaline Renard, Gavin D. Heffernan, Laura R. Jacobus, David P. Jacobus, Kurt W. Saionz, Raju Shah, Hong-Ming Shieh, Jacek Terpinski, Wenyi Zhao, Emmanuel Cornillot, Choukri Ben Mamoun

**Affiliations:** 1 Departments of Internal Medicine (Section of Infectious Diseases), Microbial Pathogenesis, and Pathology, Yale School of Medicine, New Haven, Connecticut, USA; 2 Jacobus Pharmaceutical Company Inc., Princeton, New Jersey, USA; 3 Institut de Biologie Computationnelle (IBC), and Institut de Recherche en Cancérologie de Montpellier (IRCM - INSERM U1194), Institut régional du Cancer Montpellier (ICM) & Université de Montpellier, Montpellier, France; The Children's Hospital of Philadelphia, Philadelphia, Pennsylvania, USA

**Keywords:** apicomplexan, nucleotide biosynthesis, antifolates, DHFR-TS, dihydrotriazine, antiparasitic drugs

## Abstract

*Babesia* and *Plasmodium* pathogens, the causative agents of babesiosis and malaria, are vector-borne intraerythrocytic protozoan parasites, posing significant threats to both human and animal health. The widespread resistance exhibited by these pathogens to various classes of antiparasitic drugs underscores the need for the development of novel and more effective therapeutic strategies. Antifolates have long been recognized as attractive antiparasitic drugs as they target the folate pathway, which is essential for the biosynthesis of purines and pyrimidines, and thus is vital for the survival and proliferation of protozoan parasites. More efficacious and safer analogs within this class are needed to overcome challenges due to resistance to commonly used antifolates, such as pyrimethamine, and to address liabilities associated with the dihydrotriazines, WR99210 and JPC-2067. Here, we utilized an *in vitro* culture condition suitable for the continuous propagation of *Babesia duncani, Babesia divergens, Babesia MO1,* and *Plasmodium falciparum* in human erythrocytes to screen a library of 50 dihydrotriazines and 29 biguanides for their efficacy *in vitro* and compared their potency and therapeutic indices across different species and isolates. We identified nine analogs that inhibit the growth of all species, including the *P. falciparum* pyrimethamine-resistant strain HB3, with IC_50_ values below 10 nM, and display excellent *in vitro* therapeutic indices. These compounds hold substantial promise as lead antifolates for further development as broad-spectrum antiparasitic drugs.

## INTRODUCTION

The hemoparasites *Plasmodium* and *Babesia* invade host red blood cells where they develop and multiply to cause the pathological symptoms associated with human malaria and babesiosis, respectively. While *Plasmodium* species are transmitted by mosquitoes, *Babesia* parasites are primarily transmitted by ticks. The 2023 World Health Organization report indicated that in 2022, malaria was responsible for 249 million clinical cases and 608,000 deaths worldwide, the majority of which were caused by *Plasmodium falciparum* (1). Human babesiosis has also a worldwide distribution with most cases reported in the United States where the disease is considered endemic and represents a significant risk to the blood supply (1–3). Several *Babesia* species have been shown to cause human disease (3, 4). These include *Babesia microti, Babesia duncani,* and *Babesia MO1* in the United States and *Babesia divergens* and *Babesia venatorum* in Europe and Asia (5–8). The number of reported cases of human babesiosis has increased significantly since 2011 when babesiosis became a nationally notifiable disease (2, 5, 9). The Centers for Disease Control and Prevention (CDC) recently reported that over 14,000 cases of human babesiosis were reported between 2011 and 2018 in the United States (2). Although transmission of *Babesia* parasites is primarily via the tick bite, other transmission modes have also been reported, with blood transfusion being the most common (10, 11). The disease manifestations range from asymptomatic to severe, with typical clinical symptoms including chills, fever, and sweats. For people with weak immune systems (such as cancer, lymphoma, and AIDS), immunological complications, or immune therapies, the disease can be more severe and sometimes lethal (12). The current CDC-recommended treatment protocol for human babesiosis includes combination therapy of the antimalarials azithromycin with atovaquone for mild babesiosis and clindamycin with quinine for severe babesiosis (2, 13). However, these treatments are often associated with adverse events (14–16). Consequently, persistent *Babesia* infections and recrudescence can lead to prolonged treatment (17, 18) and the emergence of mutant parasites, which are resistant to therapies (19, 20). Resistant mutations were also identified following treatment of mice infected with *B. microti* or *B. duncani* with recommended therapies (21, 22) or were selected following drug pressure *in vitro* (23, 24). Altogether, these findings highlight the need for more effective therapies for the treatment of human babesiosis.

Because folate metabolism is essential for the development and survival of parasites, targeting this process by inhibiting specific enzymatic steps in the biosynthesis of folates has long been a major focus of the antiparasitic drug development efforts for both prophylaxis and treatment (25, 26). Folate cofactors are essential in several metabolic processes including the production of purine and pyrimidine for DNA replication and amino acid metabolism. First-generation antifolates have been shown to act as competitive inhibitors of bifunctional enzyme dihydrofolate reductase thymidylate synthase (DHFR-TS) enzymes from protozoan parasites and have been used as antiparasitic drugs against *Plasmodium* and *Toxoplasma* species since World War II (25, 27, 28). The use of antifolate-based treatments is often applied when parasites become resistant to other first-line antiparasitic drugs. One such antifolate is pyrimethamine (PYM) (2,4-Diamino-5-(4-chlorophenyl)−6-ethylpyrimidine), which selectively inhibits DHFR-TS enzymes of protozoan parasites (29), and is commonly used in combination with other inhibitors targeting additional steps in the folate metabolic pathway, including sulfadoxine (drug combination known as Fansidar) and dapsone (drug combination known as Maloprim) to achieve maximum efficacy (30, 31).

A major challenge to the efficacy of antifolate-based drug therapy arose with the emergence of antifolate-resistant isolates (32, 33). Parasite lines with point mutations in the DHFR-TS enzyme showed reduced susceptibility to treatment with a combination of sulfonamide and pyrimethamine (34–36). The prevalent mutations in *P. falciparum* DHFR-TS (PfDHFR-TS) associated with resistance to pyrimethamine include N51I, C59R, S108N, and I164L. These mutations result in decreased binding affinity of antifolates to the DHFR-TS enzyme due to spatial restrictions imposed by the drug, leading to a reduction in efficacy (37, 38). *In vitro* studies and analysis of field isolates have shown that S108N is one of the most common mutations in the DHFR domain of the DHFR-TS enzyme associated with pyrimethamine resistance and is strongly associated with high levels of resistance. C59R mutation is often found in combination with S108N, contributing to increased resistance. Other mutations, including N51I, I164L, and S117N, contribute to resistance, often in combination with other mutations (25, 35, 36, 39, 40).

The small molecule WR99210 is a second-generation antimalarial triazine derivative developed in the late 1960s with improved efficacy against pyrimethamine- and chloroquine-resistant parasites (41, 42). Analysis of a library of mutants of PfDHFR-TS in *Escherichia coli* identified a set of residues in the enzyme that are associated with either sensitivity or resistance to this drug (37, 43). Despite its excellent biological activity, WR99210 exhibited poor bioavailability and caused gastrointestinal complications in healthy subjects, leading to its withdrawal from further clinical evaluation (44, 45). Third-generation biguanide prodrugs have been reported to show improved gastrointestinal tolerability and enhanced activity against malaria parasites (42, 44, 46–48). One of these, JPC-2067 showed promise when tested against an array of pathogens, including *P. falciparum, Toxoplasma gondii, Mycobacterium tuberculosis,* and *Nocardia species* (48–50). Unlike their efficacy against *Plasmodium* and *Toxoplasma* species, pyrimethamine, sulfamethoxazole, trimethoprim, and dapsone monotherapy or the combination of Pyrimethamine-sulfadoxine have been shown to have little to no activity against *B. microti* infection in Mongolian jirds (51). A moderate to effective inhibition of *in vitro* parasite growth of *Babesia gibsoni* and *Babesia bovis* was observed with trimethoprim, methotrexate, and pyrimethamine (52, 53).

Recent advances in understanding the genomic composition of various *Babesia* species that infect humans have helped identify potential targets for more effective anti-babesial drugs. Anti-babesial drug discovery was further accelerated following the development of the *B. duncani* model of infection, which combines both a continuous *in vitro* culture of the parasite in human red blood cells and infection in C3H/HeJ mice (54–58). Interestingly, previous studies have shown moderate efficacy of pyrimethamine against *B. duncani* with an IC_50_ value of 940 nM, whereas WR99210 is highly potent against the parasite with an IC_50_ value of 0.5 nM (59).

In order to search for potent but safer analogs of WR99210, we standardized the *in vitro* culture systems of several intraerythrocytic parasites in human erythrocytes in order to compare drug efficacy across different species and isolates. Using this strategy, we screened a library of 79 compounds (50 dihydrotriazines and 29 biguanides), analogs of WR99210, against *B. duncani*, *B. divergens*, *B. MO1* (*B. divergens*-like species), and *P. falciparum* drug-sensitive and -resistant strains. Nine analogs with IC_50_ values < 10 nM against all babesiosis and malaria parasites were identified and are promising early candidates for further development as broad-spectrum antiparasitic drugs.

## RESULTS

### *Babesia* DHFR-TS enzymes are suitable targets for antibabesial drug discovery

**Fig 1:**
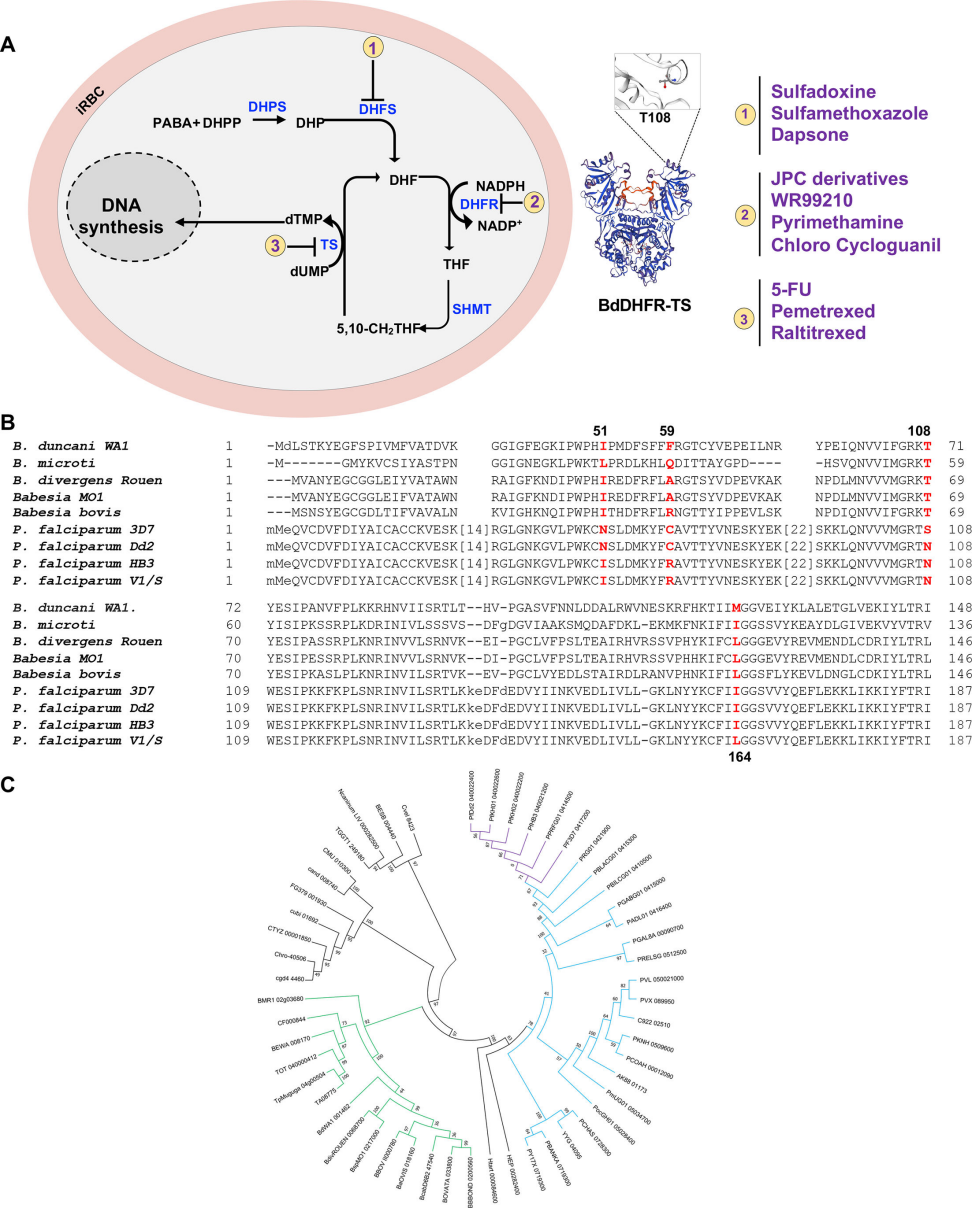
Conservation of the folate metabolic pathway among *Babesia* and *Plasmodium* parasites. (**A**) A schematic representation of folate metabolism in *Babesia* and *Plasmodium* parasites. The main enzymes (blue color) targeted by antifolates (violet color) are highlighted. Abbreviations (Abbs.): iRBC = infected red blood cell, DHP = dihydropteroate, DHPP = dihydropteroate pyrophosphate, DHPS = dihydropteroate synthase, DHF = dihydrofolate, DHFR = dihydrofolate reductase, PABA = p-aminobenzoic acid, SHMT = Serine hydroxy methyltransferase, THF = tetrahydrofolate, and TS = thymidylate synthase. (**B**) Alignment of a region of DHFR-TS enzymes from various *Babesia* species as well as pyrimethamine-sensitive (3D7) and -resistant (Dd2, HB3, and V1/S) *P. falciparum* strains. Residues known to be associated with susceptibility or tolerance to antifolates are marked in red. (**C**) Phylogenetic analysis of DHFR-TS enzymes from piroplasmids (tick-transmitted intraerythrocytic parasites) and various *Plasmodium* species. The tree was built using Phylogeny.fr pipeline combining Mafft, BMGE, and FastTree software applications. An advanced option was used to generate 1,000 bootstraps providing branch support. Clade supporting Piroplasmida sequences is shown in green; *Plasmodium* sequences are in blue, with branches of *P. falciparum* sequences represented in violet.

The availability of annotated proteomes from several human *Babesia* parasites, including *B. microti, B. duncani, B. divergens,* and *B.sp MO1*, has enabled the analysis of the primary structures of their DHFR-TS enzymes and made it possible to predict the susceptibility of these organisms to antifolates (Fig. 1A). Unlike DHFR-TS enzymes from *P. falciparum* (608 amino acids) and other *Plasmodium* species, the DHFR-TS enzymes of *Babesia* species are comparatively smaller, with sizes ranging between 502 and 514 amino acids. This size difference is largely due to the presence of insertions of 94 to 106 amino acids in most DHFR-TS enzymes of *Plasmodium* species (Fig. 1B; Fig. S1). Comparative analysis of amino acid identities and similarities within the DHFR domain revealed that the DHFR enzymes of *Babesia* species share the highest similarity with those of *P. falciparum* (59) (Fig. 1B; Fig. S1). In particular, the S108 residue associated with susceptibility to pyrimethamine in *P. falciparum* and other apicomplexan parasites corresponds to threonine in *B. microti*, *B. duncani*, *B. divergens, B. MO1,* and *B. bovis* (Fig. 1B). Phylogenetic analyses conducted on the complete protein sequences of DHFR-TS enzymes from 52 apicomplexan parasites revealed a consistent alignment with the evolutionary trajectory of species within the *Apicomplexa* phylum. The *B. microti* DHFR-TS appears as an out-group relative to other Piroplasmida (see Fig. 1C). Examination of the DHFR and TS moieties across various apicomplexan lineages revealed nearly identical evolutionary patterns. While DHFR moieties in most parasites (except *Babesia* parasites) contain insertions, no insertions or deletions were detected in the TS moieties. Furthermore, analysis of the linker region between the DHFR and TS domains (see Fig. S2) categorized them into long or short types, with the shortest linker being longer than those found in other protozoan parasites, such as kinetoplastidae (60). Taken together, these findings strongly indicate a close evolutionary relationship among DHFR-TS enzymes in *Apicomplexa*, encompassing species like *B. duncani*, *B. microti*, and *Plasmodium* spp.

### JPC-2067 inhibits parasite DHFR activity and the development of *B. duncani* in human erythrocytes

**Fig 2:**
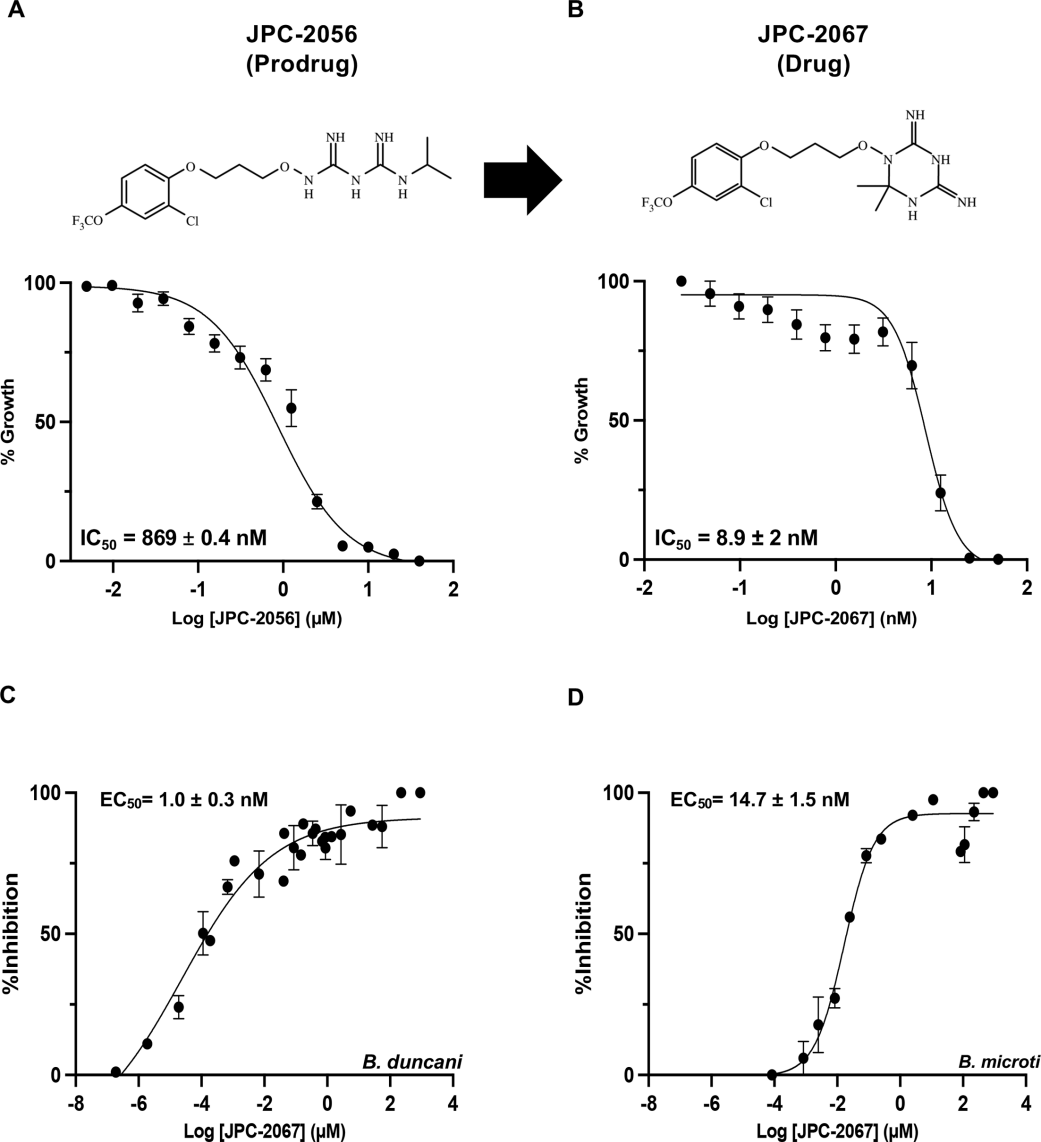
Inhibition of *B. duncani* DHFR-TS by JPC-2067 and JPC-2056. (**A and B**) Chemical structures and inhibition of *B. duncani* intraerythrocytic development by the prodrug, JPC-2056 (**A**), and its active drug JPC-2067 (**B**). The data represent the mean of two biological replicates with three technical replicates. Values represent mean ± SD. (**C-D**) A dose-dependent inhibition of purified the DHFR activity of the *B. duncani* (**C**) and *B. microti* (**D**) DHFR-TS enzymes by JPC-2067. EC_50_ values were calculated from the inhibition curves and represent mean ± SD from three independent experiments, each performed in triplicate.

The *in vitro* activity of both JPC-2067 and its pro-drug JPC-2056 (analogs of WR99210) was assessed against *B. duncani* using a continuous *in vitro* culture system in human erythrocytes. Both compounds exhibited inhibitory activity at the nanomolar level with IC_50_ values of 869.4 ± 4 nM and 8.9 ± 2 nM for JPC-2056 and JPC-2067, respectively (Fig. 2A and B). We further examined the cytotoxicity profile of both compounds against four human cell lines: HeLa, HCT-116, HepG2, and HEK-293, but no discernible effect was observed up to 900 µM. The calculated therapeutic indices were determined to be >110 for JPC-2056 and > 15,000 for JPC-2067 (Table 1).

**TABLE 1:** Cytotoxicity and therapeutic indices of JPC-2056 and its active metabolite JPC-2067

Drug	IC_50_ *B. duncani*	IC_50_ HeLa	IC_50_ HepG2	IC_50_ HEK293	IC_50_ HCT	Therapeutic index
JPC-2067	8.93 nM	>0.9 mM	1.15 mM	1 mM	1 mM	>15,000
JPC-2056	869 nM	> 0.9 mM	1 mM	1 mM	1 mM	>110

Recent studies have shown that the antifolate compound WR99210 inhibits the activity of DHFR-TS enzymes from *B. microti* and *B. duncani* (59). Therefore, we examined the inhibitory activity of JPC-2056 and JPC-2067 against the *Babesia* enzymes. The NADPH-dependent reduction of dihydrofolate (DHF) to tetrahydrofolate (THF) catalyzed by the *Babesia* enzymes was measured in the absence or presence of rising concentrations of the compounds. JPC-2067 inhibited enzyme activity with EC_50_ values of 1.0 nM and 14.7 nM for *B. duncani* and *B. microti*, respectively (Fig. 2C and D), whereas the prodrug JPC-2056 had no discernible inhibitory activity (Fig. S3A and B).

### *In vivo* efficacy of JPC-2056 in animal models of babesiosis

**Fig 3:**
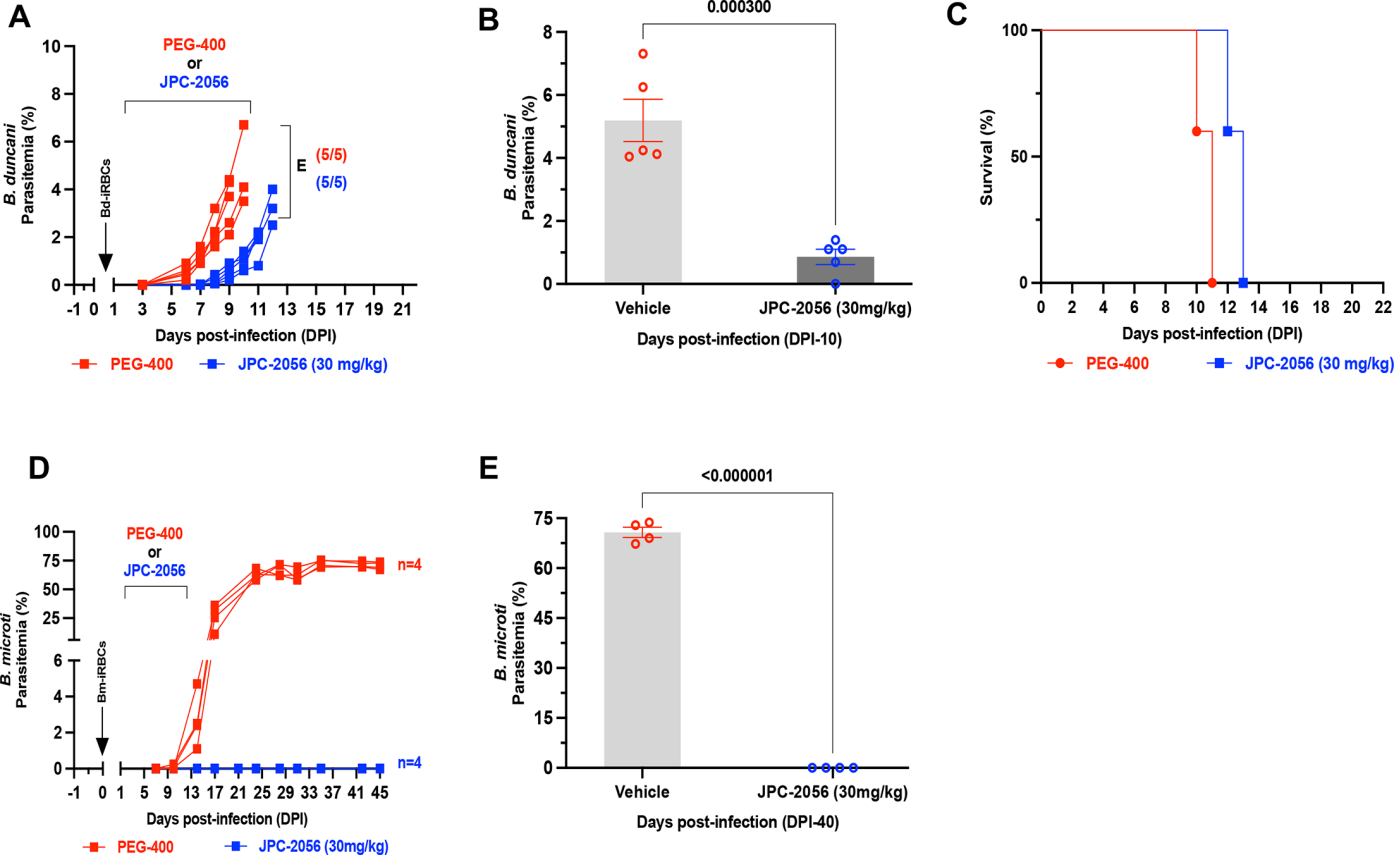
*In vivo* efficacy of JPC-2057 in mouse models of human babesiosis. (**A**) Parasitemia in female C3H/HeJ mice (*n* = 5) infected (intravenously) with 1 × 10^4^ *B. duncani*-iRBCs, which cause lethal infection in these mice, and treated with either vehicle (PEG-400) (red) or JPC-2056 (30 mg/kg, blue lines) administered via oral gavage once a day for 10 days (DPI-1 to 10). Treated mice were monitored daily and were euthanized when treatment was deemed not to be effective. Each data point depicts parasitemia measured via inspection of Giemsa-stained blood smears on the specified day. (**B**) Bar diagram depicting % parasitemia in the vehicle and JPC-2056 groups on the last day of treatment (DPI-10). Data are presented as mean ± SEM (*n* = 5 mice per group). (**C**) Kaplan-Meyer curve depicting survival of *B. duncani-*infected female C3H/HeJ mice followed by treatment with vehicle and JPC-2056. (**D**) Parasitemia in female CB17/SCID mice (*n* = 5) infected with 1 × 10^4^ *B. microti* infected RBCs (LabS1 strain) and treated with either vehicle (PEG-400, red lines) or JPC-2056 (blue lines) at 30 mg/kg once a day for 10 days. (**E**) Bar diagram showing the % parasitemia after 40 days post-infection in the vehicle and JPC-2056-treated groups. Data are presented as mean ± SEM (*n* = 5 mice per group).

The finding that JPC-2067 is effective against *B. duncani in vitro* led us to investigate the activity of its prodrug JPC-2056 in the mouse model of lethal infection (C3H/HeJ mice) (57). In this model, the mice infected with *B. duncani* parasites exhibit 100% mortality within 6–13 days post-infection (61). To evaluate the efficacy of JPC-2056, two groups of 5 female mice each were infected with 1 × 10^4^ *B. duncani*-infected erythrocytes and at day 1 post-infection (DPI-1) received either a vehicle (PEG-400) or 30 mg/kg of JPC-2056 administered orally once a day for 10 days. Mice treated with the vehicle (PEG-400) alone showed evidence of infection within 5 days and reached peak parasitemia within 10 days post-infection, with all animals succumbing to infection by DPI-10 (Fig. 3A and C). Mice treated with 30 mg/kg of JPC-2056 displayed a delay in parasite emergence and displayed reduced parasitemia over the course of the infection compared to the vehicle-treated mice. However, parasitemia in these mice continued to increase over time reaching peak levels at DPI-12 (Fig. 3A and B). These animals succumbed to lethal *B. duncani* infection at DPI-13 (Fig. 3A and C).

Building on the promising efficacy of JPC-2056 against *B. duncani* parasites *in vivo*, we extended our investigation to assess the efficacy of this compound against *B. microti* (ATCC LabS1) in the SCID (severely combined immunodeficient) mouse model of infection following inoculation with a 1 × 10^4^ infected erythrocytes. Although the control group treated with the vehicle (PEG-400) alone reached high parasitemia levels approaching 60% to 70% by 20 days post-infection (DPI-20), treatment with JPC-2056 at 30 mg/kg for 10 days (DPI-1 to 10) (Fig. 3D and E) resulted in clearance of infection with no parasites detected up to DPI-45.

### Dihydrotriazines and biguanides, analogs of JPC-2067, exhibit potent broad-spectrum antiparasitic activity *in vitro*

**Fig 4:**
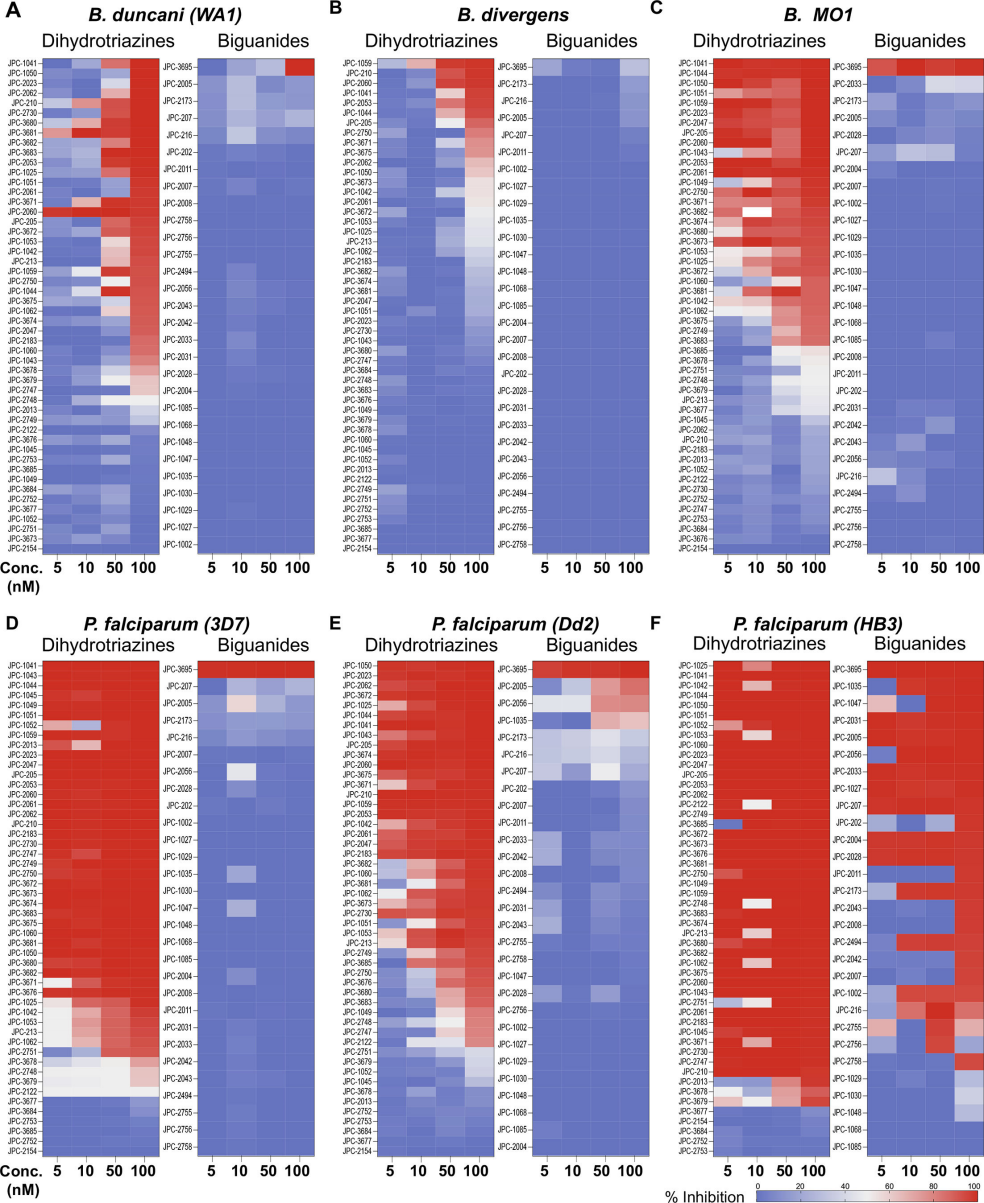
Activity of DHTs and biguanides against *Babesia* and *Plasmodium* species and isolates *in vitro*. (**A-F**) *In vitro* efficacy of dihydrotriazines and biguanides against *Babesia* [*B. duncani* WA1 (A)*, B. divergens* Rouen87 (**B**)*,* and *B.MO1* (**C**)] and *Plasmodium* species [3D7 (**D**)*,* Dd2 (**E**)*,* and HB3 (**F**)] at 5, 10, 50, and 100 nM drug concentrations. Color-coded heat maps represent mean (*n* = 3) percent inhibition of parasite growth, with dark blue representing 100% growth and dark red representing 100% inhibition.

Given the previously reported liabilities of JPC-2067 (48), we sought to evaluate the *in vitro* activity of dihydrotriazine and biguanide derivatives against several intraerythrocytic parasites for which a continuous *in vitro* culture system in human erythrocytes has been developed. Due to the diverse culture media used over the years in various laboratories for *in vitro* culture of intraerythrocytic parasites, the extrapolation of *in vitro* efficacy and other biological data from one species to another has proven to be a major hurdle in drawing meaningful conclusions across different species. It was previously shown that *B. duncani* can grow continuously in human erythrocytes in DMEM-F12 supplemented with 20% FBS (DFS20). This medium contains all the nutrients present in RPMI plus additional components including, putrescine, linoleic acid, and lipoic acid, which are essential for *B. duncani* survival. The use of DFS20 medium could thus facilitate a more precise comparison of the susceptibility of different species under similar experimental conditions (62, 63). Because *P. falciparum* is traditionally maintained in RPMI-based media, we compared the growth of the *P. falciparum* strain 3D7 in human erythrocytes in DMEM-F12- and RPMI-based culture media. Although slight differences in parasitemia were observed on both media, similar peak parasitemia levels were reached by 96h under both conditions (Fig. S4A). Using DFS20 culture conditions, we screened a total of 79 DHT derivatives (50 dihydrotriazines and 29 biguanides) (structures represented in Tables S2 and S3) against all parasites. We assessed inhibition of parasite growth at concentrations of 5, 10, 50, and 100 nM against *B. duncani* (WA-1), *B. divergens* (Rouen87), *B. MO1* (Fig. 4A through C), and *P. falciparum* (3D7, Dd2, and HB3 strains) (Fig. 4D through F). Among the compounds tested, biguanides exhibited greater efficacy against the *P. falciparum* pyrimethamine-resistant HB3 strain, but showed lesser activity against *P. falciparum 3D7* and Dd2 or *Babesia* species. In contrast, dihydrotriazines were found to be effective against both drug-sensitive and -resistant strains of *P. falciparum*, with more than 76% of the compounds tested inhibiting growth by more than 90% at 100 nM (Fig. 4D through F). The efficacy of dihydrotriazines was relatively lower against *Babesia* species, with ~34% of the compounds found to be effective against *B. duncani* (WA1 strain), *B. divergens* (Rouen87 strain), and *B. MO1* inhibiting growth by more than 90% at 100 nM. Among the 79 DHT derivatives, nine compounds (JPC-2060, JPC-3671, JPC-3681, JPC-3680, JPC-210, JPC-2748, JPC-1044, JPC-1059, and JPC-2053) were selected for their broad anti-parasitic activity and prioritized for further investigations. All nine compounds showed effective dose-dependent parasite growth inhibition with IC_50_ values ranging between 0.4 nM and 10 nM (Fig. S5; Table 2). The efficacy of these nine compounds against *P. falciparum* was similar in DFS20 and RPMI-based media (Fig. S4B; Table S3). All nine compounds were subsequently evaluated for their cytotoxicity in human cell lines (HepG2, HCT-116, HEK-293, and HeLa) and displayed low cytotoxicity. The vitro therapeutic indices for these compounds ranged between 4,000 and 100,000 (Table 2). Among these compounds, JPC-2060 emerged as the most broadly effective compound (Fig. S5), displaying high potency against *P. falciparum* strains (3D7: IC_50_ ~1.9 nM; Dd2: IC_50_ ~0.4 nM; HB3: IC_50_ ~0.6 nM), *B. duncani* WA1 strain (IC_50_ ~0.8 nM), *B. divergens Rouen87* strain (IC_50_ ~4 nM), and *B. MO1* (IC_50_ ~0.5 nM) with an *in vitro* therapeutic index of >11,000 (Table 2).

**TABLE 2:** *In vitro* efficacy and therapeutic indices of dihydrotriazine derivatives

Compound	Chemical structure	*Babesia* species			*Plasmodium falciparum*			Human cell lines	Therapeutic index
		*B. duncani* WA1 (IC_50_ in nM)	*B. divergens* Rouen87 (IC_50_ in nM)	*B. MO1* (IC_50_ in nM)	Pf3D7 (IC_50_ in nM)	PfDd2 (IC_50_ in nM)	PfHB3 (IC_50_ in nM)	Average IC_50_ (µM)	
**JPC-2060**	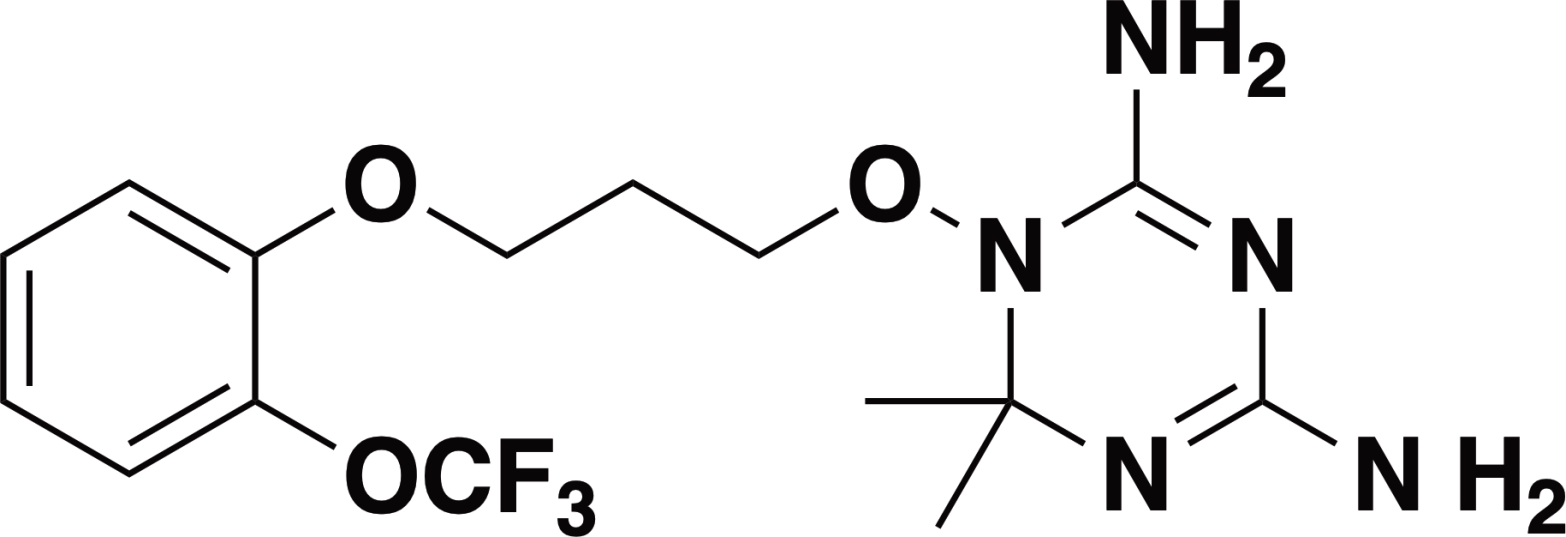	1 ± 0.06	4 ± 0.98	0.6 ± 0.07	2.3 ± 0.5	0.77 ± 0.4	0.7 ± 0.1	15	>11,000
**JPC-3671**	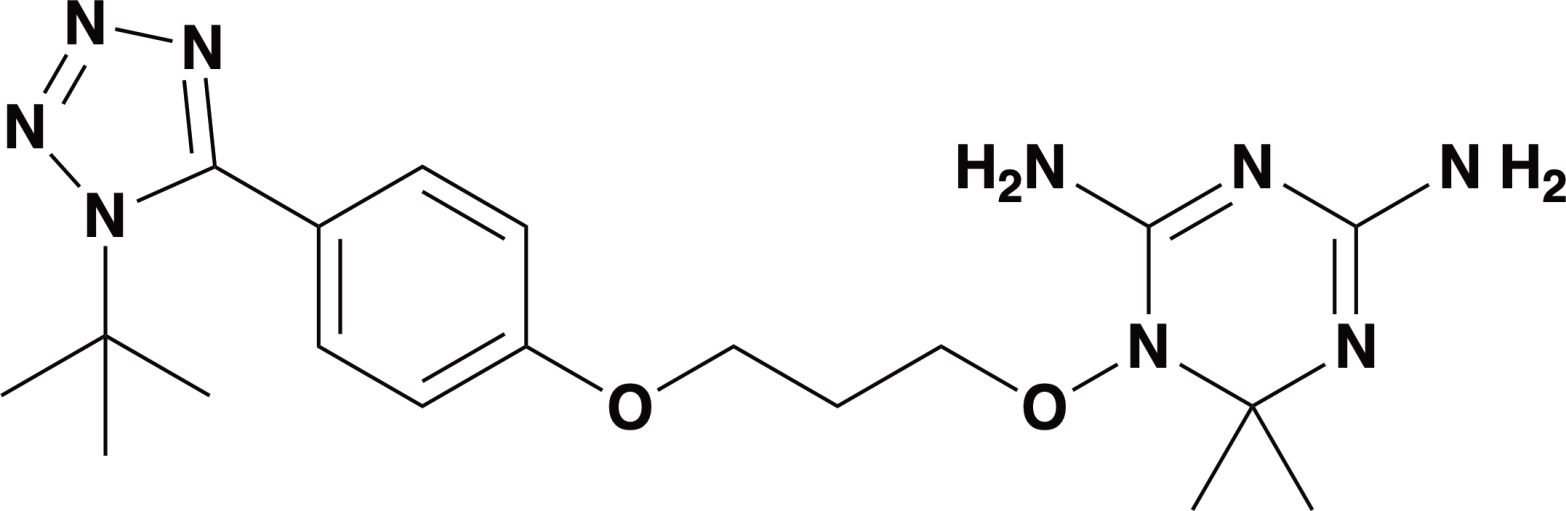	1.6 ± 0.5	24 ± 2.14	6.5 ± 0.70	1 ± 0.32	17 ± 1.5	0.4 ± 0.02	38	>19,000
**JPC-3681**	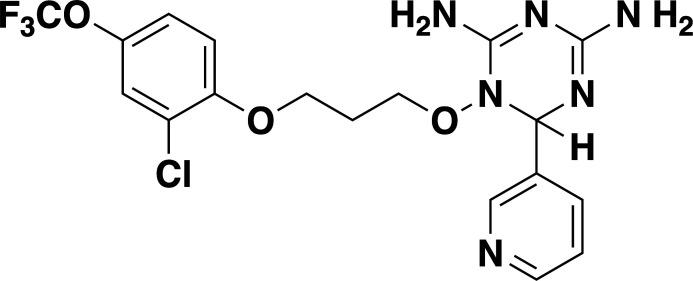	2 ± 0.20	6.4 ± 0.70	1 ± 0.07	2.3 ± 0.3	0.45 ± 0.03	0.8 ± 0.07	38	>4,000
**JPC-3680**	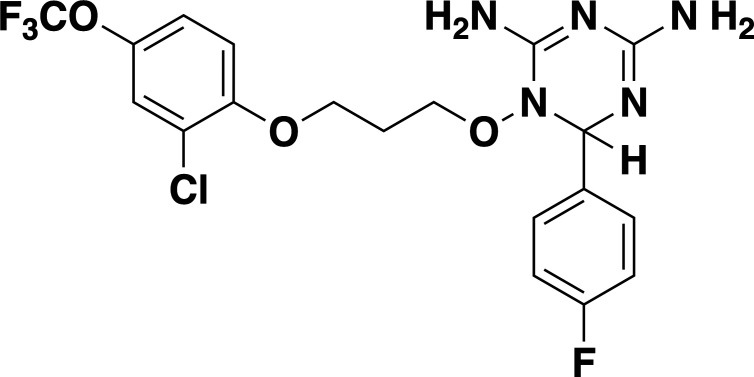	3.2 ± 0.21	40 ± 0.70	6 ± 0.5	1.7 ± 0.7	37 ± 1.25	2.1 ± 0.33	10	>600
**JPC-210**	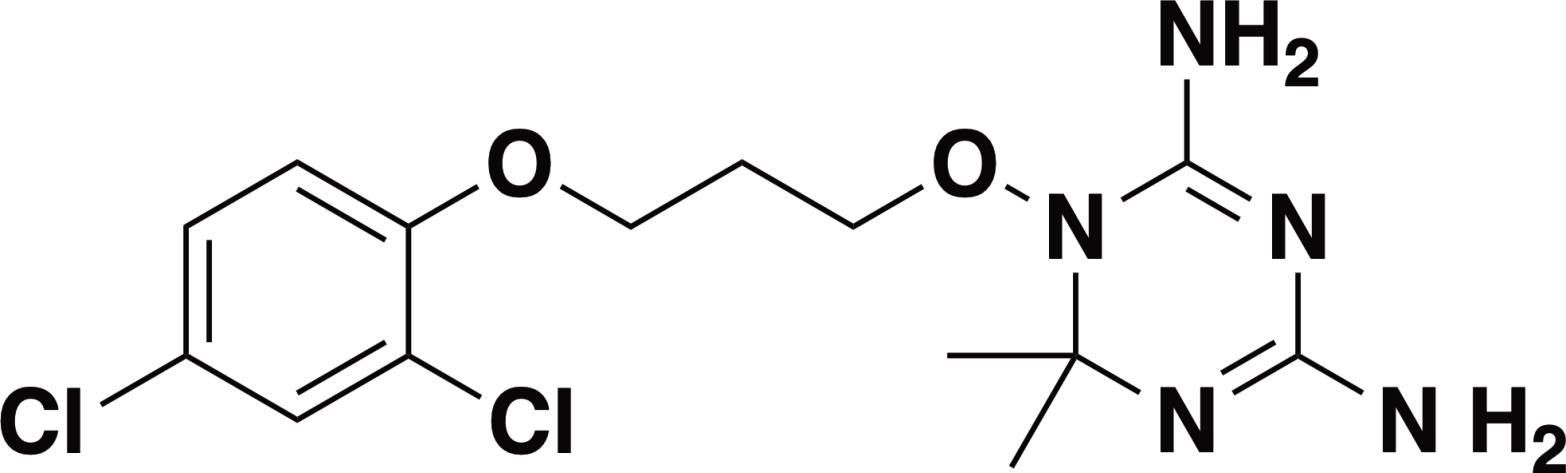	3.6 ± 0.42	3.1 ± 1.42	0.8 ± 0.24	3.1 ± 1.24	0.05 ± 0.01	0.4 ± 0.07	69	>40,000
**JPC-2748**	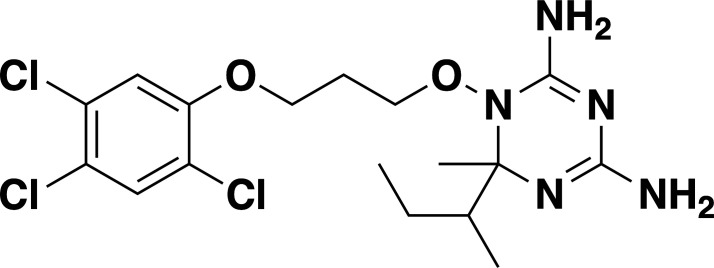	4.4 ± 0.01	3.7 ± 0.3	4 ± 0.74	3 ± 0.77	12 ± 1.13	0.41 ± 0.07	1.5	>4,000
**JPC-1044**	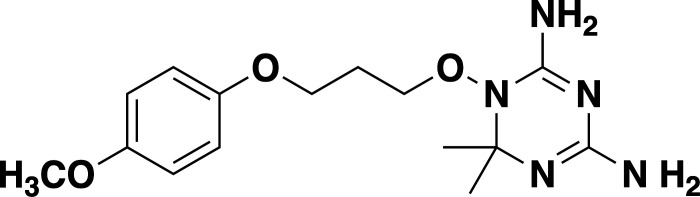	7.1 ± 0.32	12 ± 0.16	0.2 ± 0.15	3.3 ± 2	0.6 ± 0.14	0.42 ± 0.25	62	>100,000
**JPC-1059**	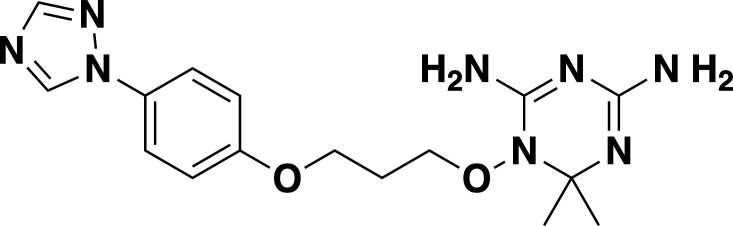	10.3 ± 0.70	3.7 ± 0.70	5.4 ± 1.4	2 ± 0.64	0.3 ± 0.02	0.3 ± 0.17	132	>100,000
**JPC-2053**	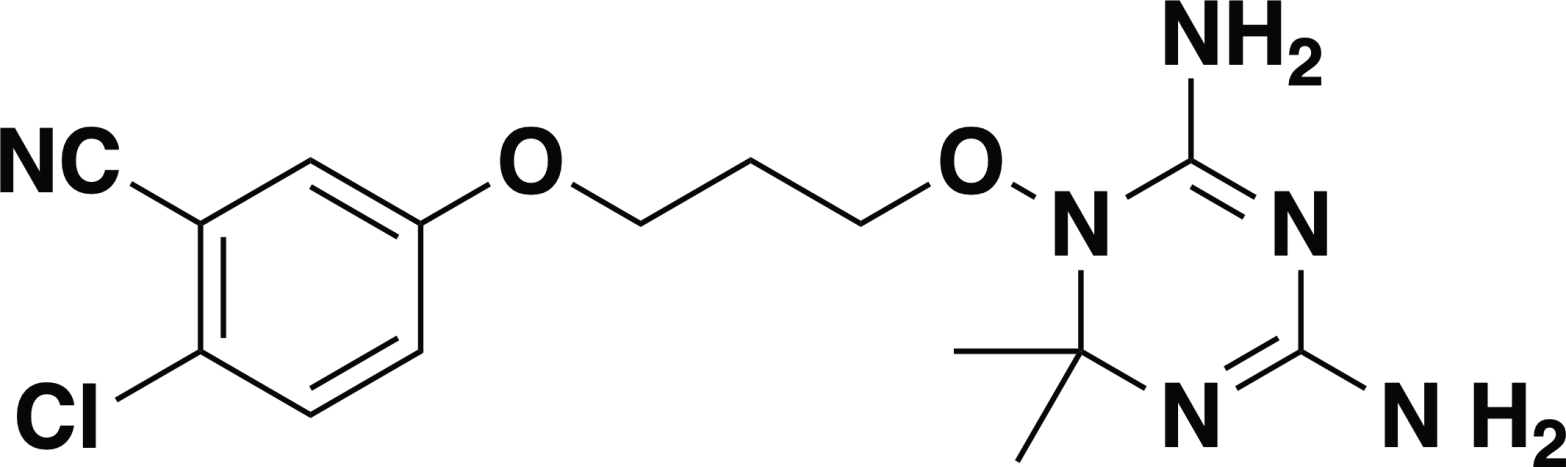	13.4 ± 1.41	6.6 ± 1.70	0.6 ± 0.50	2.6 ± 1.51	0.5 ± 0.045	0.75 ± 0.34	26	>50,000

## DISCUSSION

The DHFR-TS enzymes of *Babesia* species exhibit notable differences compared with those of *P. falciparum*, particularly in size and amino acid composition. Unlike the longer *P. falciparum* enzyme, *Babesia* DHFR-TS enzymes are comparatively smaller due to the absence of large insertions in their sequences. However, they share a high similarity with the *P. falciparum* enzyme, suggesting a close evolutionary relationship within the *Apicomplexa* phylum.

The *in vitro* assessment of the antifolate JPC-2067 and its pro-drug JPC-2056 revealed potent inhibitory activity against *B. duncani*, with nanomolar IC_50_ values. Importantly, these compounds exhibited high selectivity with minimal cytotoxicity against human cell lines. Furthermore, JPC-2067 effectively inhibited *Babesia* DHFR-TS enzymes, underscoring its mechanism of action. *In vivo* studies using JPC-2056 demonstrated efficacy of the compound against *B. duncani* in a murine model of babesiosis. Although complete parasite clearance was not achieved, treatment with JPC-2056 resulted in a significant delay in parasite burden and reduced peak parasitemia. Moreover, JPC-2056 showed remarkable efficacy against *B. microti* in a SCID mouse model of babesiosis, leading to the elimination of parasites without observable adverse effects.

Expanding on the efficacy of JPC-2067, we evaluated structurally related dihydrotriazines and biguanides for their antibabesial as well as antimalarial activity. However, comparing the efficacy of various compounds across different species and strains poses challenges in drug susceptibility testing. Variations in culture conditions can introduce confounding factors, making it difficult to draw accurate conclusions. Our present study addressed this challenge by employing DFS20, a culture medium that provides uniform nutrient supplementation, including essential elements such as putrescine, linoleic acid, and lipoic acid crucial for the growth of *B. duncani*. By utilizing DFS20 as a standardized culture medium for culturing *B. duncani*, *B. divergens*, *B. MO1*, and *P. falciparum* strains, we have created a platform conducive to more reliable cross-species and cross-strain comparisons of drug susceptibility. Using these standardized conditions, we screened a new library of 50 dihydrotriazines and 29 biguanides against all these species. Nine promising candidates were identified with IC_50_ values below 10 nM against all species and strains tested, and no toxicity at concentrations up to 900 µM. Both multi-drug-resistant and drug-sensitive *P. falciparum* strains were sensitive to the tested compounds. Among these candidates, JPC-2060 emerged as the most promising candidate for further investigation. However, treating mice infected with *B. duncani* with prodrugs for six of these candidates and all biguanides at 30 mg/kg for 5 days did not confer protection against this infection. Further optimization, including structural modifications and synthesis of more bioactive analogs, is thus necessary to improve bioavailability and extend efficacy studies to live animal models.

Our screening of analogs against *B. duncani* revealed a notable structure-activity relationship, particularly with the 2-chloro, 4-trifluoromethoxy analogs. By altering the substitution on the dihydrotriazine ring from dimethyl to aryl, we found that adding an electron-deficient aryl group at C-6 increased activity in the case of 6-(3-pyridyl) JPC-3681 and 6-(4-fluorophenyl) JPC-3680. Substituting the aryl group with an alkyl group, like 6-propyl JPC-3682, or a different pyridine, like 6-(2-pyridyl) JPC-3683, maintained some activity. Conversely, compounds with electron-rich or neutral aryl groups, such as 6-(4-methoxyphenyl) JPC-3678 and 6-phenyl JPC-3679 exhibited decreased activity. Additionally, appending nitrogen heterocycles on the terminal aryl group proved beneficial, as observed with 4-(1-(t-butyl)−1H Tetrazol-5-yl) JPC-3671 or 4-(1 H-1,2,4-triazol-1-yl) JPC-1059. These findings suggest avenues for further exploration by either combining or delving deeper into these structural regions.

In conclusion, our study underscores the importance of standardized culture conditions in facilitating accurate comparisons of drug susceptibility across different parasitic species and strains. The identification of highly potent pan-antiparasitic drugs targeting DHFR-TS enzymes represents a significant advancement in the quest for effective treatments against malaria and babesiosis. Future research should focus on further optimization of these early lead compounds to identify candidates with biological activity in animal models of babesiosis and malaria.

## MATERIALS AND METHODS

Unless otherwise stated, chemicals were purchased from commercial suppliers and used as received. WR99210, JPC- derivatives, biguanides, and DHT derivatives with purity ≥95%, as measured by reversed-phase high-performance liquid chromatography (HPLC), were procured from Jacobus Pharmaceuticals.

### Animal studies

Immunocompetent C3H/HeJ mice were procured from The Jackson Laboratory, and immunocompromised CB17/SCID mice were obtained from Envigo. All animal experiments strictly adhered to Yale University’s institutional guidelines for the care and use of laboratory animals and were conducted under the approval of a protocol sanctioned by the Institutional Animal Care and Use Committees (IACUC) at Yale University.

### Chemical synthesis of dihydrotriazines and biguanides

The dihydrotriazines were prepared as previously described by Jensen et al. (64). The majority of the dihydrotriazines were made by acidic condensation of the related pro-drug biguanide with the corresponding ketone, with acetone (Y and Z = CH_3_) as the standard as shown in step (g) as described in the scheme (Fig. S6). The biguanides were prepared as described previously and in the patent literature (65, 66). Compound identity and purity were confirmed by LC/MS. The purity of all compounds at the time of testing was >95%. The structures of the compounds assayed, the observed molecular ions, and the Chemical Abstract Service numbers (CAS #) for previously reported compounds can be found in Table S1 and S2.

### *In vitro* parasite culture of *B. duncani* and *P. falciparum*

Continuous propagation of *B. duncani* in human RBCs *in vitro* was carried out as first reported by Abraham et al. and further optimized by Singh et al., (23, 62). Briefly, parasites were cultured in a complete HL-1/DMEMF12 medium [base medium of HL-1 (Lonza, 344017)/DMEMF12 (Gibco, 11320033) supplemented with 20% heat-inactivated FBS, 2% 50X HT Media Supplement Hybrid-MaxTM (Sigma, H0137), 1% 200 mM L-Glutamine (Gibco, 25030–081), 1% 100X Penicillin/Streptomycin (Gibco, 15240-062), and 1% 10 mg/mL Gentamicin (Gibco, 15710-072)] in 5% hematocrit A+ RBCs and maintained at 37°C in a humidified chamber containing 2% O_2_, 5% CO_2_, and 93% N_2_. *P. falciparum* (3D7, Dd2, and HB3) parasites were cultured in 3%-5% human O^+^ RBCs in RPMI-1640 (Gibco, 110875093) complete medium containing 0.5% Albumax-II (Invitrogen), 2 mM L-glutamine, 50 mg/L hypoxanthine, 25 mM HEPES, 0.225% NaHCO_3_, and 10 mg/mL gentamycin. Cultures were maintained at 37°C and gassed with a sterile mixture of 4% O_2_, 5% CO_2_, and 91% N2. The culture medium was changed daily, and parasitemia was monitored by light microscope examination of Giemsa-stained thin-blood smears.

### Assessment of drug cytotoxicity on human cell lines

HeLa, HepG2, HEK293, and HCT116 cell lines were obtained from the American Type Culture Collection (ATCC) and maintained in Dulbecco’s modified Eagle’s medium (DMEM) (Invitrogen 11995–065) containing 25 mM glucose, 1 mM sodium pyruvate, and supplemented with 5 mM HEPES, 10% FBS, and penicillin-streptomycin (50 U/mL penicillin, 50 µg/mL streptomycin). Cells were seeded in a 96-well tissue culture plate (20,000 cells per well) and allowed to adhere for 24 hours, after which they were treated with a 12-step 2-fold serial dilution of each drug starting at 1 mM as the highest final concentration. Cells maintained in the medium supplemented with either 0.1% or 10% DMSO were used as negative and positive vehicle controls, respectively. The plates were incubated at 37 ˚C for 72 h, after which each well was incubated with 0.5 mg/mL of MTT reagent (MP Chemicals #02102227) for 4 h in the dark at 37 ˚C. The formazan crystals formed by living cells were solubilized with the addition of 100 µL of DMSO to each well. We measured the OD_590nm_ using a SpectraMax plate reader. From the obtained OD values, percent cell viability was estimated by normalizing to the mean of 10% DMSO wells (set as 100% toxicity) and mean of the vehicle control wells (set as 0% toxicity). Dose-response curves were plotted using GraphPad Prism version 9.1.2.

### Enzyme inhibition assays

The inhibition of DHFR activity with selected JPC derivatives was measured as previously described (59). Briefly, the purified enzyme (0.1 µM) (supporting information) (Fig. S7) was incubated with increasing concentrations of JPC-2067 or JPC-2056 in a reaction buffer containing 300 µM DHF and 300 µM NADPH. OD_340_ reduction rate (OD_340_/min) was measured for further calculation. The % inhibition was calculated using the formula:


$$=1-\frac{\left(\left(\frac{OD340}{min}\right)sample-\left(\frac{OD340}{min}\right)negative\;control\right)}{\left(\left(\frac{OD340}{min}\right)positive\;control-\left(\frac{OD340}{min}\right)negative\;control\right)}$$

The IC_50_ was determined from a sigmoidal dose-response curve using GraphPad Prism version 9.1.2.

### *In vitro* drug efficacy

The effect of DHT derivatives on the intraerythrocytic development of *P. falciparum, B. duncani*, *B. divergens* and *B. MO1* and the determination of IC_50_ values were conducted as previously reported (54, 67). Briefly, the parasites cultured *in vitro* (0.1% parasitemia with 5% hematocrit in complete DFS20 medium or RPMI-1640) were treated with various concentrations of the compounds in a 96-well plate and incubated at 37 ˚C for either 60 h in the case of *B. duncani* or 72 h in the case of *P. falciparum.* Parasitemia was then determined using the SYBR Green-I method by adding an equal volume of parasite culture to the lysis buffer (0.008% saponin, 0.08% Triton-X-100, 20 mM Tris-HCl (pH = 7.5), and 5 mM EDTA) containing SYBR Green-I (0.01%). The mixture was then incubated at 37°C for 1 h in the dark. The fluorescence was measured at 480 nm (excitation) and 540 nm (emission) using a BioTek Synergy™ Mx Microplate Reader. Background fluorescence from uninfected erythrocytes maintained in a complete DFS20 and RPMI-1640 medium was subtracted from each sample, and the 50% inhibitory concentration (IC_50_) of the drug was determined by plotting drug concentrations versus parasite growth using GraphPad Prism 9.2.1. Data are shown as mean ± SD from two biological replicates with three technical replicates.

### *In vivo* drug efficacy

Both C3H/HeJ and CB17/SCID mice aged 5–6 weeks (4 to 5 mice per group in each of the studies described herein) were injected intravenously with (1 × 10^4^ iRBCs) of *B duncani* (WA-1) and *B. microti* (LabS1) parasites, respectively. The infected mice were treated by oral gavage over 10 days (DPI −1 to 10). During the treatment, each mouse received 100 µL of either vehicle (PEG-400) or prodrug JPC-2056 (30 mg/kg). Percent parasitemia was determined by counting at least 3,000 RBCs from Giemsa-stained thick blood smears.

### Statistical analysis

Data sets were analyzed with GraphPad Prism version 9.1.2. Statistical significances were determined using unpaired *t*-tests.

## SUPPLEMENTAL MATERIAL

Data Set S1 aac.00423-24-s0001.xlsx Gene IDs, species names, proteins, and protein lengths.

Supplemental figures aac.00423-24-s0002.tiff Figures S1 to S7.

Supplemental text aac.00423-24-s0003.docx Supplemental methods; legends for Fig. S1 to S7.

Table S1 aac.00423-24-s0004.docx List of dihydrotriazines and structures.

Table S2 aac.00423-24-s0005.docx List of biguanides and structures.

Table S3 aac.00423-24-s0006.docx *In vitro* efficacy (IC50 values) of DHTs against *P. falciparum* (3D7) in different media conditions.

ASM does not own the copyrights to Supplemental Material that may be linked to, or accessed through, an article. The authors have granted ASM a non-exclusive, world-wide license to publish the Supplemental Material files. Please contact the corresponding author directly for reuse.
