# Supplementary figures and images for: In vitro efficacy of next-generation dihydrotriazines and biguanides against babesiosis and malaria parasites
Source: Antimicrob Agents Chemother. 2024 Aug 13;68(9):e00423-24. doi: 10.1128/aac.00423-24 (PMC11373198; doi:10.1128/aac.00423-24)

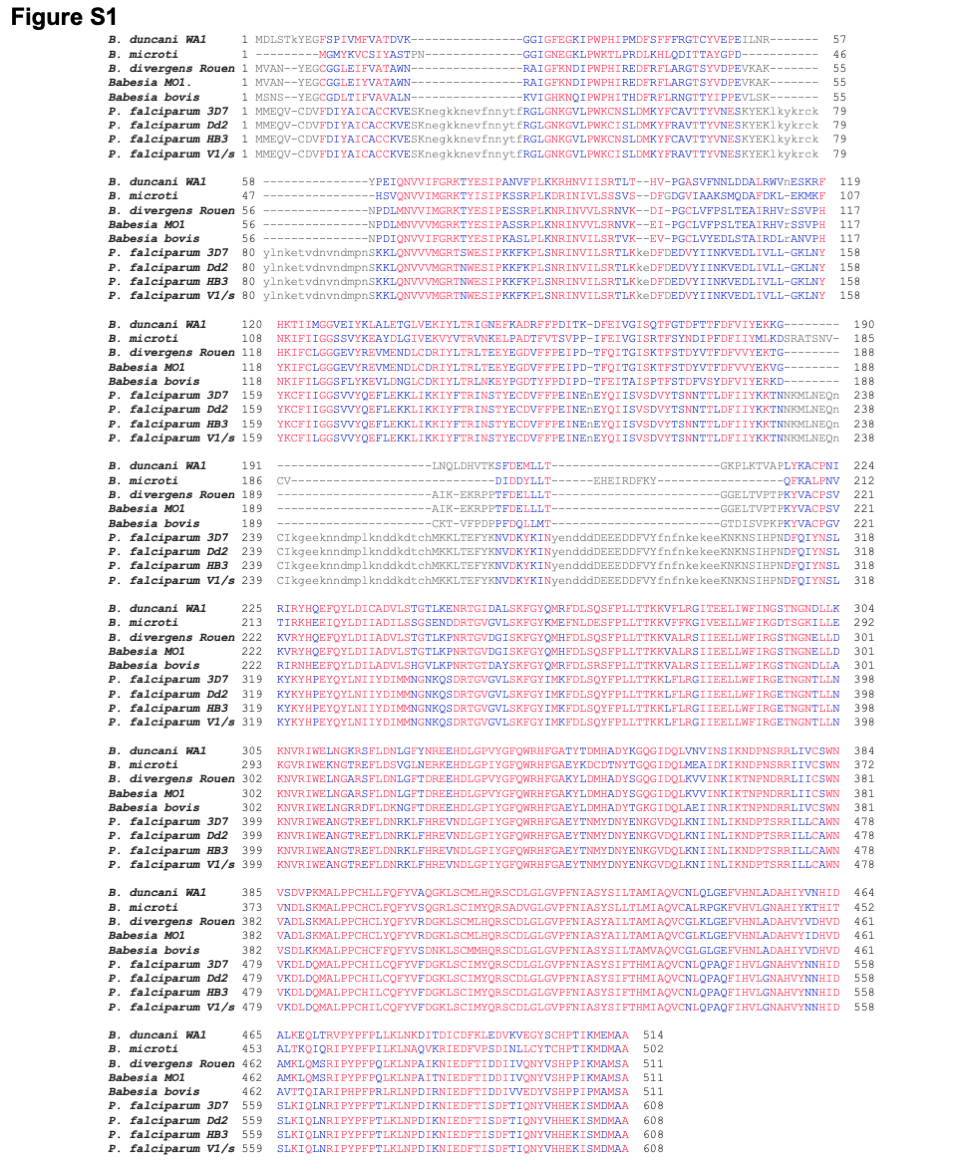

Supplement: Supplemental figures — Figures S1 to S7. [file aac.00423-24-s0002.tiff]
