# Supplementary material for: In vitro efficacy of next-generation dihydrotriazines and biguanides against babesiosis and malaria parasites
Source: Antimicrob Agents Chemother. 2024 Aug 13;68(9):e00423-24. doi: 10.1128/aac.00423-24 (PMC11373198; doi:10.1128/aac.00423-24)
Supplement: Supplemental text — Supplemental methods; legends for Fig. S1 to S7. [file aac.00423-24-s0003.docx]

**Supplemental Methods:**

**Culture conditions and drug efficacy in different culture media.** To create uniform conditions for evaluating pan-antiparasitic drug efficacy, we assessed the growth of *P. falciparum* in RPMI-1640 and various DMEM-F12 conditions. Parasites initially cultured in RPMI-1640 + Albumax-II were washed with incomplete media and then propagated in DMEM-F12 media supplemented with 20% and 10% heat-inactivated FBS, RPMI-1640 with 10% human serum, and RPMI-1640 with 0.5% Albumax-II at 5% hematocrit using A+ RBCs. Cultures were maintained at 37°C in a humidified chamber with 2% O2, 5% CO2, and 93% N2. Drug efficacy and IC_50_ determinations were assessed under these conditions

***Cloning, Expression, and Purification of recombinant B. duncani and B. microti DHFR-TS enzymes***

Dihydrofolate reductase-thymidylate synthase (DHFR-TS) gene from *B. duncani* (*Bd*DHFR-TS BdWA1_001462) and *B. microti* (*Bm*DHFR-TS BMR1_02g03680) were cloned into pMAL-c4x by GenScript USA Inc. Both enzymes were expressed as fusion proteins with N’-terminal maltose binding protein (MBP) tag. Constructs of pMAL-c4x-*Bd*DHFR-TS and pMAL-c4x-*Bm*DHFR-TS were transformed by heat shock into *E. coli* BL-21(DE3)**.** *E. coli* BL-21(DE3) harboring pMAL-c4x-DHFR-TS was grown overnight in 2 ml LB ampicillin (100 µg/mL). Cultures were diluted 100-fold in fresh LB medium with 0.2% glucose and grown to A_600_ of ~0.5 at 37°C. DHFR-TS expression was induced by the addition of 0.5 mM isopropyl thiogalactoside (IPTG) followed by growth for overnight at 16°C or at room temperature (RT). The cells were harvested by centrifugation (8,000 × g × 10 min, 4°C), washed by resuspension in water, and re-centrifuged. Cells were used directly for purification or kept frozen at -20°C. Prior to enzyme purification, cells were re-suspended in binding buffer containing 25 mM Tris-HCl pH 8, 500 mM NaCl, 0.5% glycerol, and 50mM L-arginine. Cells were supplemented benzonase DNAse (250U/µl) and 3-((3-cholamidopropyl) dimethylammonio)-1-propanesulfonate (CHAPS) (0.002%) and were disrupted by sonication on ice using Omni Sonic Ruptor 400 Ultrasonic Homogenizer (15-sec burst at 70% amplitude, 3 times, with 30-sec cooling intervals). A soluble supernatant was prepared by centrifugation (16,000 × g × 20 min) of the cells sonicate.

Recombinant DHFR-TSs were purified from the clear cell extract supernatant using amylose resin (NEB), methotrexate-agarose beads, and in some cases size exclusion chromatography (SEC). Briefly, soluble supernatant was incubated with amylose beads in binding buffer with gentle agitation for 1h at 4°C. Following enzyme adsorption, the affinity matrix was transferred to a column and unbound proteins were removed by washing with binding buffer. Purified enzymes were eluted with elution buffer containing 10 mM maltose. Amylose elution fractions were loaded onto MTX-agarose resin and purified DHFR-TS enzymes were eluted using 2 mM dihydrofolic acid (DHF). Maltose or DHF residuals were removed by dialysis or using a PD-10 column (GE Healthcare) and the Purified protein stocks were then adjusted to 25% glycerol ^v^/_v_, aliquoted, flash-frozen, and stored at -80˚C.

**Figure legends:**

**Figure S1.** Alignment of the full-length protein sequences of DHFR-TS from *Babesia* and *Plasmodium* parasites.

**Figure S2.** Alignment of the sequence linking the DHFR and TS domains in the DHFR-TS bifunctional enzymes from 52 apicomplexan parasites (ID, species name, protein length are provided in Suppl Data).

**Figure S3. Inhibition of *B. duncani and B. microti* DHFR-TS activity by JPC-2056.** A dose-dependent inhibition of the DHFR activity of purified DHFR-TS enzymes from *B. duncani* **A**) and *B. microti* **B**) by JPC-2056. EC_50_ values were calculated from the inhibition curves and represent mean ± SD from three independent experiments, each performed in triplicate.

**Figure S4.** **In vitro efficacy of the top 9 DHTS** **against *P. falciparum (3D7)* in different culture conditions. A)** Depicted bar diagrams are total fluorescence readings from synchronized *P. falciparum (3D7)* parasites (0.5% parasitemia) cultured continuously for two generations (96 hours). The SYBR Green-I based parasite quantification was conducted on parasites grown in DFS-20% FBS (Black), DFS12-10% FBS (Brown), RPMI1640-10% Human serum (Green), and RPMI1640-albumax-II (Blue) conditions. **B)** Growth of *P. falciparum* in the absence or presence of increasing concentrations of DHTs in DFS20 (Red) and RPMI1640-Human serum (Black) culture conditions. For each tested compound, IC_50_ curves in graphs depict n=2 in triplicates; error bars indicate ± standard error of the mean (SEM).

**Figure S5. Efficacy of shortlisted DHT derivatives.** Dose-response sigmoidal semilogarithmic graphs with parasite growth inhibition (y-axis) versus the log concentration of 9 DHT derivatives (x-axis) against *Babesia* (*B. duncani WA1*(green)*, Bdiv Rouen87* (blue)*, and B. spMO1* (brown)) and *Plasmodium* species (3D7 (red), Dd2 (yellow), and HB3 (purple)).

**Figure S6. Scheme for the chemical synthesis of dihydrotriazines and biguanides**. Reagents: (a) 1,3-dibromopropane (for n = 3), NaOH, tetrabutylammonium hydrogen sulfate; (b) AcNHOH, NaOH, or KOH; alcoholic solvent; (c) concentrated HCl, MeOH; (d) dicyandiamide, aqueous EtOH, heat, and then aqueous NaOH to neutralize; (e) sodium dicyanamide, HCl, alcoholic solvent, heat; (f) EtOAc, heat; (g) HCl, MeOH; (h) room temp, DMF.

**Figure S7.** Coomassie blue-stained SDS-PAGE gels showing recombinant *B. duncani* **(A)** and *B. microti* -DHFR-TS **(B)** enzymes purified from E. coli cell extracts using an amylose resin. Total cell extracts (CE), flow-through (FT), and elution fractions along with protein standard marker (M) were loaded on a gradient SDS-PAGE (4-10%). The purified Bd and Bm-DHFR-TS fused with the MBP-tag migrate at approximately ~90 kDa size. Purified enzymes were eluted with elution buffer containing 10 mM maltose. Amylose elution fractions were loaded onto MTX-agarose resin and purified DHFR-TS enzymes were eluted using 2 mM dihydrofolic acid (DHF). Maltose or DHF residuals were removed by dialysis or using a PD-10 column (GE Healthcare).

**Table S1** List of Dihydrotriazine derivatives with Chemical Abstract System (CAS) number, functional groups, and corresponding references.

**Table S2** List of Biguanides with Chemical Abstract System (CAS) number, functional groups and corresponding references

**Table S3** In vitro efficacy ( IC_50_ values) of DHTs against *P falciparum (3D7)* in different media conditions
