# Supplementary material for: In vitro efficacy of next-generation dihydrotriazines and biguanides against babesiosis and malaria parasites
Source: Antimicrob Agents Chemother. 2024 Aug 13;68(9):e00423-24. doi: 10.1128/aac.00423-24 (PMC11373198; doi:10.1128/aac.00423-24)
Supplement: Table S1 — List of dihydrotriazines and structures. [file aac.00423-24-s0004.docx]

**Table S1.** List of Dihydrotriazine derivatives with Chemical Abstract System (CAS) number, functional groups, and corresponding references.

| **Compound Identifier** | **R** | **X** | **n** | **Y_1_, Z** | **Exact**  **Mass** | **Obs.**  **Mol. Ion**  **(M+H)^+^** | **Salt Form** | **CAS Number** | **Reference** |
| --- | --- | --- | --- | --- | --- | --- | --- | --- | --- |
| **JPC-1025** | 4-Br | O | 3 | CH_3,_ CH_3_ | 369.08  371.08 | 370.4  372.4 | HCl | 381247-66-1 | (1) |
| **JPC-1041** | *2-Naphthyl | O | 3 | CH_3,_ CH_3_ | 341.19 | 342.5 | HCl | 381247-84-3 | (1) |
| **JPC-1042** | 4-NO_2_ | O | 3 | CH_3,_ CH_3_ | 336.15 | 337.4 | HCl | 30737-46-3 | (2) |
| **JPC-1043** | 4-F | S | 3 | CH_3,_ CH_3_ | 325.41 | 326.4 | HCl | 381247-78-5 | (1) |
| **JPC-1044** | 4-OCH_3_ | O | 3 | CH_3,_ CH_3_ | 321.18 | 322.4 | HCl | 381247-68-3 | (1) |
| **JPC-1045** | 2,4,5-tri-Cl | O | 2 | CH_3,_ CH_3_ | 379.04  381.03 | 380.2  382.3 | HCl | 381247-80-9 | (1) |
| **JPC-1049** | 4-t-Butyl | O | 3 | CH_3,_ CH_3_ | 347.23 | 348.5 | HBr | 30712-19-7 | None |
| **JPC-1050** | *2-(6-Br-Naphthyl) | O | 3 | CH_3,_ CH_3_ | 419.10  421.09 | 420.4  422.4 | HBr | 871125-54-1^fb^ | (3) |
| **JPC-1051** | 4-Cl | O | 4 | CH_3,_ CH_3_ | 339.15 | 340.4 | HBr | 30712-33-5 | (4) |
| **JPC-1052** | 4-Cl | SO_2_ | 3 | CH_3,_ CH_3_ | 373.10 | 374.4 | HBr | 1348523-36-3 ^fb^ | None |
| **JPC-1053** | 3,4-di-Cl | O | 3 | CH_3,_ CH_3_ | 359.09 | 360.4 | HCl | 179183-68-7 ^fb^ | (5, 6) |
| **JPC-1059** | 4-N-Triazole | O | 3 | CH_3,_ CH_3_ | 358.19 | 359.5 | HBr | 871125-56-3 ^fb^ | (3) |
| **JPC-1060** | 4-Cl | CH_2_ | 3 | CH_3,_ CH_3_ | 323.15 | 324.4 | HCl | 1350088-09-3 ^fb^ | None |
| **JPC-1062** | 4-SO_2_CH_3_ | O | 3 | CH_3,_ CH_3_ | 369.15 | 370.5 | HCl | None | None |
| **JPC-2013** | 4-Cl-Phenyl | None | None | CH_3,_ CH_3_ | 251.09 | 252.2 | HCl | 152-53-4 | 4-Cl-Cycloguanil |
| **JPC-2023** | 4-OCF_3_ | O | 3 | CH_3,_ CH_3_ | 375.15 | 376.5 | HCl | 701976-23-0 | (7) |
| **JPC-2047** | 4-CF_3_ | O | 3 | CH_3,_ CH_3_ | 359.16 | 360.5 | HBr | 849926-87-0 | (3, 6) |
| **JPC-205** | 4-F | O | 3 | CH_3,_ CH_3_ | 309.16 | 310.4 | HCl | 381247-65-0 | (1) |
| **JPC-2053** | 3-CN, 4-Cl | O | 3 | CH_3,_ CH_3_ | 350.13 | 351.4 | HCl | None | None |
| **JPC-2060** | 2-OCF_3_ | O | 3 | CH_3,_ CH_3_ | 375.15 | 376.5 | HCl | 849926-89-2 ^fb^ | (6) |
| **JPC-2061** | 3-OCF_3_ | O | 3 | CH_3,_ CH_3_ | 375.15 | 376.5 | HCl | None | None |
| **JPC-2062** | 4-OCH2CF_3_ | O | 3 | CH_3,_ CH_3_ | 389.17 | 390.5 | HCl | None | None |
| **JPC-210** | 2,4-Cl | O | 3 | CH_3,_ CH_3_ | 359.09  361.09 | 360.4  362.4 | HCl | 381247-71-8 | (1) |
| **JPC-2122** | 4-O(4-OCF_3_-Ph) | O | 3 | CH_3,_ CH_3_ | 467.18 | 468.5 | HCl | 871125-57-4 ^fb^ | (3) |
| **JPC-2183** | 3-OCF_3_,4-Cl | O | 3 | CH_3,_ CH_3_ | 409.11 | 410.4 | HCl | 871125-58-5 ^fb^ | (3) |
| **JPC-213** | 4-Cl | O | 3 | CH_3,_ CH_3_ | 325.13 | 326.4 | HCl | 381247-64-9 | (1) |
| **JPC-2730** | 2,4,5-tri-Cl | O | 3 | CH_3_, iPr | 421.08  423.08 | 422.4  424.4 | HCl | None | None |
| **JPC-2747** | 2,4,5-tri-Cl | O | 3 | CH_3_, iPrCH_2_ | 435.10  437.10 | 436.4  438.4 | HCl | None | None |
| **JPC-2748** | 2,4,5-tri-Cl | O | 3 | CH_3_, EtCHCH_3_ | 435.10  437.10 | 436.5  438.5 | HCl | None | None |
| **JPC-2749** | 2,4,5-tri-Cl | O | 3 | CH_3_, Bu | 435.10  437.10 | 436.4  438.4 | HCl | None | None |
| **JPC-2750** | 2,4,5-tri-Cl | O | 3 | Spiro C_5_H_10_ | 433.08  435.08 | 434.4  436.4 | HCl | 381247-83-2 | (1) |
| **JPC-2751** | 2,4,5-tri-Cl | O | 3 | Spiro C_5_H_8_  4’-(CH_3_)_2_ | 461.12  463.12 | 462.5  464.5 | HCl | None | None |
| **JPC-2752** | 2,4,5-tri-Cl | O | 3 | Spiro Adamantyl | 485.12  487.11 | 486.5  488.5 | None | None | None |
| **JPC-2753** | 2,4,5-tri-Cl | O | 3 | Spiro Norbornane | 445.08  447.08 | 446.5  448.5 | None | None | None |
| **JPC-3671** | 4-C-(N_1_-tBu tetrazole) | O | 3 | CH_3,_ CH_3_ | 415.24 | 416.5 | None | None | None |
| **JPC-3672** | 4-(BuOC=O) | O | 3 | CH_3,_ CH_3_ | 391.22 | 392.5 | HCl | None | None |
| **JPC-3673** | 4-(4-OCF_3_-Ph-) | O | 3 | CH_3,_ CH_3_ | 451.18 | None | HCl | None | None |
| **JPC-3674** | 4-(4-OCH_3_-Ph-) | O | 3 | CH_3,_ CH_3_ | 397.21 | None | HCl | None | None |
| **JPC-3675** | 2-Ph | O | 3 | CH_3,_ CH_3_ | 367.20 | None | HBr | 494785-88-5 ^fb^ | None |
| **JPC-3676** | 4-(3-CF_3_,4-ClPh-) | O | 3 | CH_3,_ CH_3_ | 469.15 | None | HBr | None | None |
| **JPC-3677** | 2-Cl, 4-OCF_3_ | O | 3 | CH_3_ (NHAc) | 451.12 | None | None | None | None |
| **JPC-3678** | 2-Cl, 4-OCF_3_ | O | 3 | H, 4-OCH_3_-Ph | 487.12 | None | HCl | None | None |
| **JPC-3679** | 2-Cl, 4-OCF_3_ | O | 3 | H, Ph | 457.11 | None | HCl | None | None |
| **JPC-3680** | 2-Cl, 4-OCF_3_ | O | 3 | H, 4-F-Ph | 475.10 | None | HCl | None | None |
| **JPC-3681** | 2-Cl, 4-OCF_3_ | O | 3 | H, 3-Pyridyl | 458.11 | None | HCl | None | None |
| **JPC-3682** | 2-Cl, 4-OCF_3_ | O | 3 | H, Propyl | 423.13 | None | HCl | None | None |
| **JPC-3683** | 2-Cl, 4-OCF_3_ | O | 3 | H, 2-Pyridyl | 458.11 | None | HCl | None | None |
| **JPC-3684** | Triazolo, Triazine  4-ClPh | None | None | NA | 261.05 | None | HCl FB | 2226513-93-3 ^fb^ | (8) |
| **JPC-3685** | 4-Ph | O | 3 | CH_3,_ CH_3_ | 367.20 | None | HCl | None | None |
| **2154-68** | Triazolo, Triazine  4-ClBn  (See JPC- 3684) | None | None | NA | 275.07 | None | HCl | None | None |
| **2154-69** | 2-Cl, 4-OCF_3_ | O | 3 | H, 4-CF_3_-Ph | 525.10 | None | HCl | None | None |

^fb^: The corresponding CAS number is for the Free Base, not the salt.

*: Direct Connection to the X-Propyloxy chain.

**REFERENCES:**

1. Jensen NP, Ager AL, Bliss RA, Canfield CJ, Kotecka BM, Rieckmann KH, Terpinski J, Jacobus DP. 2001. Phenoxypropoxybiguanides, prodrugs of DHFR-inhibiting diaminotriazine antimalarials. J Med Chem 44:3925-31.

2. Jung.E., V. V, G. C. 1968. 1,2-Dihydro-1,3,5-triazine derivatives, processes for their production and their use for the production of medicinal preparations.

3. Hunt SY, Detering C, Varani G, Jacobus DP, Schiehser GA, Shieh HM, Nevchas I, Terpinski J, Sibley CH. 2005. Identification of the optimal third generation antifolate against P. falciparum and P. vivax. Mol Biochem Parasitol 144:198-205.

4. Turnbull M, Crowley J, Chrystal E, Clough J, Youle D, Beautement K, Barnett S, Ponsford R, Outred D, Mamalis P, Hatter G, Smith P, Bellini P. 1996. Fungicidal compositionWO9615672.

5. Turnbull M, Crowley J, Chrystal E, Clough J, Youle D, Beautement K, Barnett S, Ponsford R, Outred D, Mamalis P, Hatter G, Smith P, Bellini P. 1996. Herbicides.

6. Shearer TW, Kozar MP, O'Neil MT, Smith PL, Schiehser GA, Jacobus DP, Diaz DS, Yang YS, Milhous WK, Skillman DR. 2005. In vitro metabolism of phenoxypropoxybiguanide analogues in human liver microsomes to potent antimalarial dihydrotriazines. J Med Chem 48:2805-13.

7. Jacobus D, Schiehser G, Shieh H-M, Jensen N, Terpinski J. 2004. Biguanide and Dihydrotriazine Derivatives

8. Hassan AY, Sarg MT, Bayoumi AH, El-Deeb MA. 2018. Synthesis and Anticancer Evaluation of Some Novel 5-Amino[1,2,4]Triazole Derivatives. Journal of Heterocyclic Chemistry 55:1450-1478.
