# Supplementary material for: In vitro efficacy of next-generation dihydrotriazines and biguanides against babesiosis and malaria parasites
Source: Antimicrob Agents Chemother. 2024 Aug 13;68(9):e00423-24. doi: 10.1128/aac.00423-24 (PMC11373198; doi:10.1128/aac.00423-24)
Supplement: Table S2 — List of biguanides and structures. [file aac.00423-24-s0005.docx]

**Table S2.** List of Biguanides derivatives with Chemical Abstract System (CAS) number, functional groups, and corresponding references.

| **Compound Identifier** | **R** | **X** | **n** | **Y_2_** | **Exact**  **Mass** | **Obs.**  **Mol. Ion**  **(M+H)^+^** | **Salt Form** | **CAS Number** | **Reference** |
| --- | --- | --- | --- | --- | --- | --- | --- | --- | --- |
| **JPC-1002** | 4-Cl | O | 3 | iPr | 327.15 | 328.4 | HCl | 756887-29-3 | (1-3) |
| **JPC-1027** | 4-Br | O | 3 | iPr | 371.10  373.09 | 372.4  374.4 | HCl | 381247-36-5 ^fb^ | (1) |
| **JPC-1029** | H | (Ethyl) | (Ethyl) | H | 205.13 | 206.2 | HCl | 834-28-6 | Phenformin |
| **JPC-1030** | None | None | 3 | H | 129.10 | 130.2 | HCl | 1115-70-4 | Metformin |
| **JPC-1035** | 2,4-Cl | O | 3 | iPr | 361.11  363.10 | 362.4  364.4 | HCl | 381247-44-5 ^fb^ | (1) |
| **JPC-1047** | 3,4-Cl | O | 3 | iPr | 361.11  363.10 | 362.4  364.4 | HCl | 756887-43-1 | (1-5) |
| **JPC-1048** | 4-tBu | O | 3 | iPr | 349.25 | 350.5 | HCl | 2651213-25-9 | (1) |
| **JPC-1068** | 4-Cl | O | 3 | CH_2_CF_3_ | 367.10 | None | None | None |  |
| **JPC-1069** | 4-Cl | O | 3 | CH2CCH | 323.11 | 324.4 | HCl | None |  |
| **JPC-1085** | 4-Cl | O | 3 | Cyclopropyl | 325.13 | 326.4 | HCl | None |  |
| **JPC-1086** | 4-Cl | O | 3 | CH_2_ Cyclopropyl | 339.15 | 340.4 | HCl | None |  |
| **JPC-2004** | 4-SO_2_CH_3_ | O | 3 | iPr | 371.16 | 372.5 | Succinate | None |  |
| **JPC-2005** | 4-OCF_3_ | O | 3 | iPr | 377.17 | 378.5 | HCl | 701976-18-3 ^fb^ | (4-6) |
| **JPC-2007** | 4-CF_3_ | O | 3 | iPr | 361.17 | 362.5 | Succinate | 849926-84-7 | (4, 5) |
| **JPC-2008** | 3,4-Cl | O | 3 | iPr | 361.11  363.10 | 362.4  364.4 | Succinate | None |  |
| **JPC-2011** | 4-(4-Cl-Ph- SO_2_) | O | 3 | iPr | 467.14 | 468.5 | None | None |  |
| **JPC-202** | 4-F | O | 3 | iPr | 311.18 | 312.4 | Succinate | 381247-35-4 ^fb^ | (1) |
| **JPC-2028** | 4-OCF_3_ | O | 3 | Cyclopropyl | 375.15 | 376.5 | Sulfate | 701976-20-7 ^fb^  701976-21-8  HCl | (4-6) |
| **JPC-2031** | 4-Me-Tetrazole | O | 3 | iPr | 375.21 | 376.5 | None | None |  |
| **JPC-2033** | 4-CN | O | 3 | Cyclopropyl | 316.16 | 317.4 | None | None |  |
| **JPC-2042** | 4-OCF_3_ | O | 3 | H | 335.12 | 336.4 | Succinate | 701976-39-8 ^fb^ | (6) |
| **JPC-2043** | 3,4-Cl | O | 3 | H | 319.06  321.06 | 320.2  322.2 | Succinate | None |  |
| **JPC-2056** | 2-Cl, 4-OCF_3_ | O | 3 | iPr | 411.13 | 412.5 | None | 701976-24-1 | (4-6) |
| **JCP-207** | 3-Cl | None | None | iPr | 253.11 | 254.2 | None | 807308-41-4 | (7, 8) |
| **JPC-216** | 2-Cl | None | None | iPr | 253.11 | 254.2 | HCl | 878792-89-3 | (7, 8) |
| **JPC-2173** | 2,4-Cl  5-OCF_3_ | O | 3 | iPr | 445.09  447.09 | 446.4  448.5 | None | None |  |
| **JPC-2494** | 2-Cl, 4-OCF_3_ | O | 3 | H | 369.08 | 370.4 | None | None |  |
| **JPC-2755** | 2-Cl, 4-OCF_3_ | O | 3 | t-Bu | 425.14 | 426.5 | None | None |  |
| **JPC-2756** | 2-Cl, 4-OCF_3_ | O | 3 | Adamantyl | 503.13 | 504.5 | None | None |  |
| **JPC-2758** | 4-Cl | O | 3 | H | 285.10 | 286.4 | HCl | 2972313-55-4 ^fb^ |  |

^fb^: The corresponding CAS number is for the Free Base, not the salt.

*: Direct Connection to the X-Propyloxy chain.

**REFERENCES:**

1. Jacobus D, Jensen N. 2003. Antimalarial N,N'-substituted biguanides derived from hydroxylaminesUS20030040544.

2. Jensen NP, Ager AL, Bliss RA, Canfield CJ, Kotecka BM, Rieckmann KH, Terpinski J, Jacobus DP. 2001. Phenoxypropoxybiguanides, prodrugs of DHFR-inhibiting diaminotriazine antimalarials. J Med Chem 44:3925-31.

3. Parenti MD, Pacchioni S, Ferrari AM, Rastelli G. 2004. Three-dimensional quantitative structure-activity relationship analysis of a set of Plasmodium falciparum dihydrofolate reductase inhibitors using a pharmacophore generation approach. J Med Chem 47:4258-67.

4. Patel DS, Ramesh M, Bharatam PV. 2012. CytochromeP450 isoenzyme specificity in the metabolism of anti-malarial biguanides: molecular docking and molecular dynamics analyses. Medicinal Chemistry Research 21:4274-4289.

5. Shearer TW, Kozar MP, O'Neil MT, Smith PL, Schiehser GA, Jacobus DP, Diaz DS, Yang YS, Milhous WK, Skillman DR. 2005. In vitro metabolism of phenoxypropoxybiguanide analogues in human liver microsomes to potent antimalarial dihydrotriazines. J Med Chem 48:2805-13.

6. Jacobus D, Schiehser G, Shieh H-M, Jensen N, Terpinski J. 2004. Biguanide and Dihydrotriazine Derivatives

7. Curd FH, Hendry JA, et al. 1948. Synthetic antimalarials; an alternative route to N1-aryl-N5-alkyldiguanides. J Chem Soc:1630-6.

8. M. Bami HL, PC. G. 1949. Studies in Antimalarials Part X. N1-Aryl-N5-heterocyclic-biguanides. . Journal of the Indian Institute of Science 31A, II: 9-14.
