# Supplementary material for: In vitro efficacy of next-generation dihydrotriazines and biguanides against babesiosis and malaria parasites
Source: Antimicrob Agents Chemother. 2024 Aug 13;68(9):e00423-24. doi: 10.1128/aac.00423-24 (PMC11373198; doi:10.1128/aac.00423-24)
Supplement: Table S3 — In vitro efficacy (IC50 values) of DHTs against P. falciparum (3D7) in different media conditions. [file aac.00423-24-s0006.docx]

**Table S3.** In vitro efficacy ( IC_50_ values) of DHTs against *P falciparum (3D7)* in different media conditions**.**

|  | ***P.falciparum* 3D7**  **(IC_50_ in nM)** | |
| --- | --- | --- |
| **Compound** | **RPMI-1640 + 10%human serum** | **DFS20** |
| JPC-2060 | 0.8 ± 0.17 | 1.9 ± 0.29 |
| JPC-3671 | 2.6 ± 0.02 | 2.1 ± 0.016 |
| JPC-3681 | 1.0 ± 0.08 | 0.7 ± 0.072 |
| JPC-3680 | 1.8 ± 0.30 | 1.2 ± 0.030 |
| JPC-210 | 1.2 ± 0.10 | 1.6 ± 0.025 |
| JPC-2748 | 2.0 ± 0.061 | 0.2 ± 0.12 |
| JPC-1044 | 3.8 ± 0.022 | 2.0 ± 0.02 |
| JPC-1059 | 1.4 ± 0.021 | 1.3 ± 0.09 |
| JPC-2053 | 1.4 ± 0.021 | 1.5 ± 0.071 |
